# Draft genome sequence of a KPC-2-producing, meropenem-durlobactam non-susceptible *Klebsiella pneumoniae* ST11-KL64 clinical isolate

**DOI:** 10.1128/mra.00046-26

**Published:** 2026-03-02

**Authors:** Hongxia Wen, Yu Feng, Zhiyong Zong

**Affiliations:** 1 Laboratory for Pathogen Research, West China Hospital, Sichuan University 12530, Chengdu, China; 2 Center of Infectious Diseases, West China Hospital, Sichuan University 12530, Chengdu, China; 3 Division of Infectious Diseases, State Key Laboratory of Biotherapy 429364, Chengdu, China; Rochester Institute of Technology, Rochester, New York, USA

**Keywords:** *Klebsiella pneumoniae*, ST11-KL64, meropenem-durlobactam, non-susceptibility

## Abstract

We report the draft genome of a clinically isolated *Klebsiella pneumoniae* ST11-KL64 strain 143027 that is non-susceptible to the meropenem-durlobactam combination (minimum inhibitory concentration [MIC], 256/4 μg/mL). This genome sequence provides a foundation for investigating reduced susceptibility to emerging carbapenem-β-lactamase inhibitor combinations used to treat infections caused by carbapenem-resistant *K. pneumoniae*.

## ANNOUNCEMENT

There is a growing reliance on next-generation β-lactamase inhibitors against carbapenem-resistant *Klebsiella pneumoniae* (CRKP) (1). Durlobactam, a diazabicyclooctane β-lactamase inhibitor with activity against a broad spectrum of Ambler class A, C, and D β-lactamases, has emerged as a promising partner in carbapenem-based regimens (2). We report the draft genome of an isolate exhibiting a high MIC of meropenem in the presence of durlobactam.

Strain 143027 was isolated in 2025 from the urine of an adult patient with a urinary tract infection as part of routine care at our hospital. The strain was streaked onto blood agar and incubated at 37°C overnight. A single colony subcultured in Luria-Bertani broth overnight was preliminarily identified as *K. pneumoniae* by matrix-assisted laser desorption ionization time-of-flight mass spectrometry (Bruker Daltonics, Bremen, Germany). The strain was resistant to piperacillin-tazobactam, cefotaxime, ceftazidime, cefepime, aztreonam, imipenem, meropenem, ertapenem, doripenem, amikacin, ciprofloxacin, nitrofurantoin, trimethoprim-sulfamethoxazole, and tigecycline using VITEK 2 (bioMérieux, Marcy-l’Étoile, France) and polymyxin B (MIC, 8 mg/L) using Clinical and Laboratory Standards Institute (CLSI) broth microdilution (3). MIC of meropenem-durlobactam was determined using CLSI broth microdilution with a fixed 4 mg/L durlobactam (3). The strain exhibited a high MIC (256/4 mg/L) of meropenem-durlobactam, indicating its non-susceptibility.

For whole-genome sequencing, genomic DNA was extracted after overnight culture in Luria-Bertani broth using the QIAamp DNA Blood Mini Kit (Qiagen, Hilden, Germany) according to the manufacturer’s instructions. A sequencing library targeting 300-bp insert size was prepared using the NEBNext Ultra II DNA Library Prep Kit (New England Biolabs, Ipswich, MA), and sequencing was performed on an Illumina NovaSeq 6000 platform (San Diego, CA) with 150-bp paired-end reads. In total, 5,876,608 read pairs were generated (approximately 1.76 GB of raw data), corresponding to an estimated 298× genome coverage. Unless otherwise specified, all bioinformatics analyses were performed using default parameters. Raw reads were quality trimmed with Trimmomatic v0.39 (4), and reads longer than 50 bp were randomly subsampled to 150-fold coverage using SeqKit v2.8.2 (5). We assembled the subsampled reads *de novo* with SPAdes v4.2.0 (6) in isolate mode, yielding a draft genome of 174 contigs totaling 5,917,162 bp, with an N*50* of 176,099 bp and a G+C content of 56.88%. The assembled genome was automatically annotated upon submission using the NCBI Prokaryotic Genome Annotation Pipeline v6.10 (7), which identified 5,824 protein-coding genes, 162 pseudogenes, and 11 noncoding RNAs. Average nucleotide identity analysis with FastANI v1.34 (8) confirmed it as *K. pneumoniae*, showing 98.91% identity to type strain ATCC 13883^T^ (accession no. CP064368). Genotyping with Kleborate v3.2.4 (9) assigned it to sequence type ST11 and capsular type KL64. AMRFinderPlus v4.2.5 (10) identified genes mediating resistance to aminoglycosides (*rmtB1*), carbapenems (*bla*_KPC-2_), other β-lactams (*bla*_CTX-M-65_, *bla*_LAP-2_, *bla*_SHV-11_, and *bla*_TEM-1_), chloramphenicol (*catA2*), fosfomycin (*fosA*), fluoroquinolones (*qnrS1*), sulfonamides (*sul2*), tetracycline (*tet(A*)), and trimethoprim (*dfrA14*). These were concordant with the phenotypic resistance profile.

The mechanism mediating meropenem-durlobactam non-susceptibility remains undetermined. Nevertheless, this genome provides a precious resource for comparative genomics to identify potential resistance mechanisms.

## Data Availability

The draft genome sequence of strain 143027 has been deposited in GenBank under accession number JBTIXW000000000. Illumina raw sequencing reads have been submitted to the Sequence Read Archive (SRA) under accession number SRR36602811.
